# Degradation of Li/S Battery Electrodes On 3D Current Collectors Studied Using X-ray Phase Contrast Tomography

**DOI:** 10.1038/srep10921

**Published:** 2015-06-04

**Authors:** L. Zielke, C. Barchasz, S. Waluś, F. Alloin, J.-C. Leprêtre, A. Spettl, V. Schmidt, A. Hilger, I. Manke, J. Banhart, R. Zengerle, S. Thiele

**Affiliations:** 1 Laboratory for MEMS Applications, Department of Microsystems Engineering - IMTEK, University of Freiburg, Georges-Koehler-Allee 103, 79110 Freiburg, Germany; 2 French Atomic Energy and Alternative Energy Agency (CEA), Laboratory of Innovation for New Energy Technologies and Nanomaterials (LITEN), 17 Rue des Martyrs, 38054 Grenoble Cedex 9, France; 3 University Grenoble Alpes, LEPMI, F-38000 Grenoble, France; 4 CNRS, LEPMI, F-38000 Grenoble, France; 5 Institute of Stochastics, Ulm University, Helmholtzstr. 18, 89069 Ulm, Germany; 6 Helmholtz Zentrum Berlin, Hahn-Meitner-Platz 1, 14109 Berlin, Germany; 7 HSG-IMIT Institut für Mikro- und Informationstechnik der Hahn-Schickard-Gesellschaft e.V., Georges-Koehler-Allee 103, 79110 Freiburg, Germany; 8 FIT, University of Freiburg, Stefan-Meier-Straße 21, 79104 Freiburg, Germany

## Abstract

Lithium/sulphur batteries are promising candidates for future energy storage systems, mainly due to their high potential capacity. However low sulphur utilization and capacity fading hinder practical realizations. In order to improve understanding of the system, we investigate Li/S electrode morphology changes for different ageing steps, using X-ray phase contrast tomography. Thereby we find a strong decrease of sulphur loading after the first cycle, and a constant loading of about 15% of the initial loading afterwards. While cycling, the mean sulphur particle diameters decrease in a qualitatively similar fashion as the discharge capacity fades. The particles spread, migrate into the current collector and accumulate in the upper part again. Simultaneously sulphur particles lose contact area with the conducting network but regain it after ten cycles because their decreasing size results in higher surface areas. Since the capacity still decreases, this regain could be associated with effects such as surface area passivation and increasing charge transfer resistance.

Rechargeable lithium-ion (Li-ion) batteries have been under extensive research over the past 20 years, owing to the increasing energy consumption of electronic devices^1^. Lithium transition metal oxides are currently dominating the commercial Li-ion battery market. This is due to their advantages, such as high energy density, high operating voltage, and low self-discharge. However, achievable gravimetric energy densities are known to be limited to 200-250 Whkg^−1^ for these technologies, which is not sufficient to meet the battery requirements for extended ranges of electric vehicles^2^.

The lithium/sulphur (Li/S) technology is one of the most promising candidates for next-generation energy storage systems, featuring a high theoretical capacity of 1675 mAhg^−1^. This is much larger than the 100-250 mAhg^−1^ capacity attainable with the conventional Li-ion positive electrode materials. The discharge potential is around 2.15 V, and the complete Li/S system should allow for reaching a gravimetric energy density of 500 Whkg^−1^. In addition, elemental sulphur is readily available and non-toxic. These advantages should allow high energy batteries to be produced cheaply and safely^3^.

Lithium metal reacts with elemental sulphur (S_8_) to produce lithium polysulphides of composition Li_2_S_n_ (2 ≤ n ≤ 8)^4^. First, long polysulphides chains form, such as Li_2_S_8_ and Li_2_S_6_. During discharge, the polysulphide chain length is shortened, as the sulphur is being further reduced, and intermediate species such as Li_2_S_4_ and Li_2_S_2_ are produced. At the end of discharge, the final product is lithium sulphide (Li_2_S). The overall reaction is:


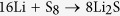


While sulphur, Li_2_S, and Li_2_S_2_ compounds are poorly soluble in organic electrolytes, the intermediate polysulphides (Li_2_S_n_ with 3 ≤ n ≤ 8) dissolve into the electrolyte during discharge and the electrochemical reaction takes place in solution^5^. On the contrary, at the end of charge and discharge, sulphur-based compounds solidify again, and either sulphur or Li_2_S_2_/Li_2_S is deposited onto the positive electrode surface^6^.

The Li/S technology has attracted the attention of the electrochemistry community for many years^7^,^8^,^9^,^10^,^11^. However, this quite promising system has not been yet commercialized, because practical performances still fall short of expectations: limited practical discharge capacities are obtained, while both the cyclability and coulombic efficiency are rather low.

The reasons for low sulphur utilization and capacity fading are still unclear. Some authors claim that the dissolved polysulphides are responsible for lithium metal corrosion and Li_2_S deposition on the negative electrode^11^,^12^. Other publications ascribe capacity fading to changes of the state of sulphur active material (solid/soluble) during electrochemical reaction and to the changes of sulphur electrode morphology during cycling^13^,^14^. Some studies gathered evidence for passivation phenomenon of the sulphur electrode over cycling, caused by the deposition of solid and insulating S_8_ and Li_2_S_2_/Li_2_S products, at the end of charge or discharge respectively^15^. They demonstrated that these insulating products are responsible for cathode insulation and blocking, and that they prevent the electrochemical reaction from proceeding further^5^,^10^,^16^. Finally, some authors claim that the capacity fading is related to the irreversible deposition of Li_2_S material on the positive surface during cycling, which then accumulates on the electrode and does not further participate in the electrochemical reaction afterwards^14^,^15^,^16^,^17^. Owing to this controversial and abundant literature on the capacity fading of Li/S technology, it appears crucial to understand in greater depth the degradation phenomena of the Li/S cells, and more specifically of sulphur electrodes.

The electrode morphology has a pronounced influence on battery degradation. As such, different strategies towards positive electrode composition can be found in literature to improve the Li/S cell electrochemical performances by changing the composition of the positive electrode. Sulphur and lithium polysulphides can be confined into nanostructured materials^11^,^12^,^18^,^19^. These authors report a high discharge capacity along with an improved cyclability. However, they finally point out that lithium polysulphides progressively diffuse into the electrolyte, thus leading to changes of the morphology of the positive electrode^11^. Other strategies rather refer to the use of alternative positive electrode compositions, using, for example, alternative current collectors such as metallic foams or carbon nanotubes^20^,^21^,^22^,^23^. Previous work reported that the discharge capacity of Li/S systems is governed by the solubility of lithium polysulphides and the positive electrode passivation^10^,^13^,^16^. It was also demonstrated that the discharge capacity is linked to the morphology of the positive electrode and its specific surface area.

In summary, the study of morphological changes is crucial to understand degradation phaenomena in Li/S batteries. Therefore we applied three-dimensional synchrotron X-ray phase contrast tomography. Use of this method for the investigation of degradation of Li/S batteries has never been reported. However, Zhou *et al.* applied X-ray tomography to investigate individual parts of their Li/S battery in means of porosity, surface structure and confined sulphur after 50 cycles^24^. Nelson *et al.* used synchrotron two dimensional X-ray transmission microscopy to investigate their sulphur battery electrodes, in operando, during different stages of one cycle^25^. They found, that the sulphur particle size decreases during this cycle. Their two-dimensional observation could be confirmed and extended in our three-dimensional studies of sulphur particle parameter evolution during various cycle steps and in a representative volume. In contrast to conventional lab tomography systems, the highly coherent monochromatic synchrotron X-rays in combination with the phase contrast technique are more suited to investigate actual particle sizes, contact areas and small features, like cracks in the sulphur particles. This is because attenuation coefficients are well defined and no beam hardening effect occurs, which again influences segmentation quality and discriminability of the different phases, which is important when it comes to quantify contact areas. In the past, X-ray tomography has also been applied to the exploration of the microstructure and degradation of other types of batteries such as alkaline batteries or lithium-based electrodes^26^,^27^,^28^,^29^.

Here, we report the use of X-ray phase contrast tomography to extract morphological parameters influencing degradation of a Li/S cell, and more specifically of the positive sulphur electrode. In contrast to two-dimensional imaging techniques, this method allows to access large, representative volumes. In these volumes three-dimensional parameters like contact areas between the phases, size distributions, and spatial distributions throughout the whole electrode height can be quantified in dependence of the aging state of the battery electrode.

## Results

For this study, different types of current collector morphologies were investigated with respect to their electrochemical response. Among the best suited materials^22^, a Li/S electrode based on non-woven carbon current collector (NWC) was then investigated at different stages of ageing using X-ray phase contrast tomography. We identify morphological changes of the sulphur phase which could be responsible for the strong capacity fading of these kinds of electrodes.

### Electrochemical response

**Figure 1:**
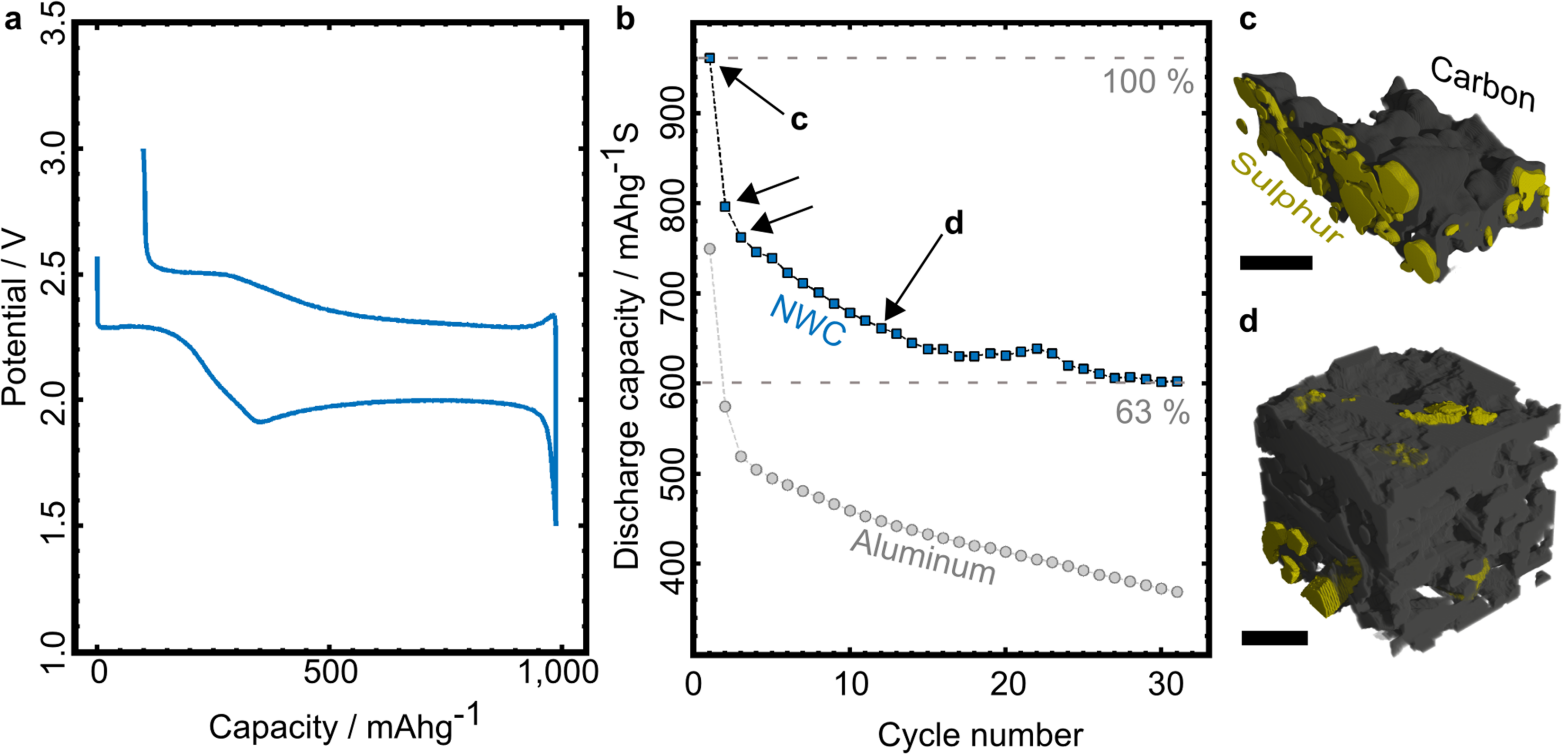
Electrochemical response of an NWC-based Li/S electrode and an aluminum foil-based Li/S electrode. (**a**) Charge/discharge profile of a sulphur electrode on NWC current collector. (**b**) Discharge capacity fading over 31 cycles of an electrode with an NWC current collector (squares) and an aluminum foil current collector (spheres). The electrode with the NWC current collector has higher discharge capacity throughout cycling. Black arrows mark the electrodes which we investigated in this study. (**c**)- (**d**) Examples of morphological changes in the electrode found with X-ray phase contrast tomography (c: before the first cycle, d: before the 11^th^ cycle). Sulphur loading strongly decreases, the particles shrink, spread and partially loose contact to the carbon materials of the electrode, namely the current collector and the carbon- binder domain. The black size markers are 50 μm wide.

The electrochemical response (Fig. 1a) of positive sulphur electrodes on NWC current collectors was evaluated in coin cells. The resulting voltage profile exhibits two plateaus (Fig. 1a) which is consistent with literature^4^. In Fig. 1b, the discharge capacities of an electrode on a NWC current collector and on an aluminum foil are shown. The discharge capacity of the electrode with the NWC current collector is (223 ± 11) mAhg^−1^ higher on average, but still shows a marked decrease in capacity during cycling. Therefore, we focus on the superior NWC based electrode in the following X-ray tomography study.

The first plateau in Fig. 1a at 2.3 V vs. Li^+^/Li can be attributed to the reduction of elemental sulphur to long chain lithium polysulphides (Li_2_S_n_ 4 ≤ n ≤ 8), while the longer but lower voltage plateau relates to the reduction of medium chains, formation of short-chain lithium polysulphides (Li_2_S_n_ 2 ≤ n ≤ 3), and of the final discharge product Li_2_S. During charging, reverse processes occur and elemental sulphur is deposited on the electrode, associated by a change of its crystalline form from α-sulphur to β-sulphur (metastable phase)^6^. The initial discharge capacity of the electrode on NWC is about 970 mAhg^−1^, which corresponds to an initial practical loading of about 3.9 mAh cm^−2^.

The discharge mechanism of the Li/S system -consisting in precipitation/dissolution cycles of active material- is held responsible for the capacity fading observed in Fig. 1 (only 63% of the initial capacity is retained after 31 cycles).

In the following section, we show that X-ray phase contrast tomography is an excellent tool to clearly differentiate between sulphur and carbon in the electrodes.

### X-ray tomographic reconstruction

**Figure 2:**
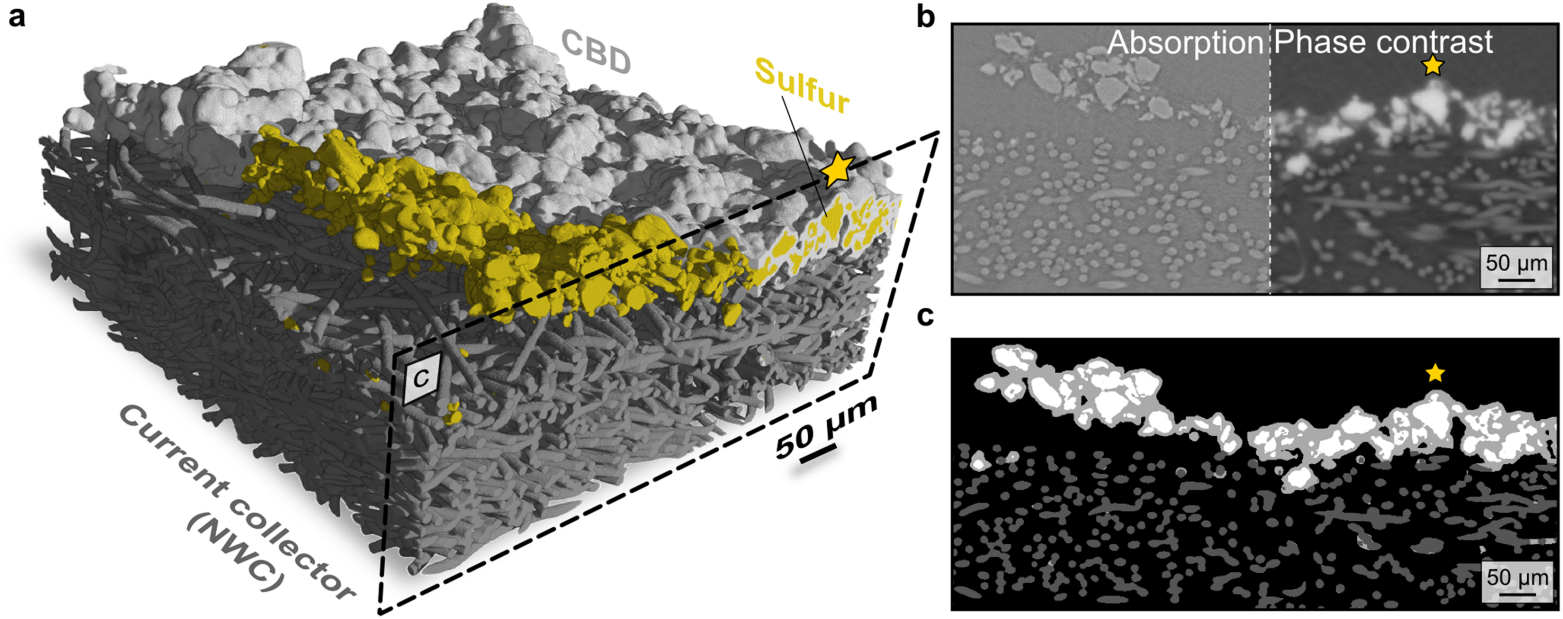
Three-dimensional representation of an uncycled Li/S electrode. (**a**) 3D representation of an uncycled sulphur electrode on NWC. The carbon-binder domain (CBD) is only partially depicted (light grey) to emphasize the sulphur phase distribution. The representation contains ~900 aligned images such as the one shown in c. (**b**) X-ray tomogram showing absorption contrast (left side) and phase contrast (right). For segmentation, the information from each of these two types of images was combined. The resulting segmentation is shown in c. (**c**) Segmented image. Sulphur is depicted in white, the CBD in light grey and the fibres of the NWC in dark grey. The sulphur is almost fully coated by the CBD. For orientation we marked the same particle with an orange star in a, b and c.

Four X-ray phase contrast tomography reconstructions of sulphur electrodes on NWC were obtained: i) an uncycled electrode, ii) an electrode after 1 cycle, iii) after 2 cycles and iv) after 10 cycles. This is because the electrochemical data in Fig. 1b shows that the most severe changes in discharge capacity occur in the first 10-15 cycles. Especially the electrode morphology after the first and second cycle is of interest, since here the discharge capacity fading is not gradual but rather abrupt. In Fig. 2a, we present a three-dimensional geometrical representation of the uncycled sulphur electrode on NWC. It consists of approx. 900 aligned images, containing quadratic pixels with a length of 876 nm each. The original resolution of the tomography is 438 nm per pixel, but we coarsed the data once for better handling. For the uncycled electrode the in-plane dimensions are 750 μm x 770 μm with a thickness of 340 μm. The sulphur phase (yellow) is almost completely covered by a mixture of carbon and binder (light grey), which is the reason for only partially depicting the carbon-binder domain (CBD) in Fig. 2a. Before the first cycle, the sulphur is almost completely on top of the NWC.

Regarding representativeness, the largest sulphur particle found in all electrodes investigated fits at least 12 times into the smallest in-plane direction of all investigated electrodes. Also all samples are completely imaged regarding the through-plane direction. Therefore, our observations concerning sulphur particles can be considered representative.

To be able to investigate the ageing process in these kinds of electrodes, a reliable segmentation had to be done. The methods are described in the methods section. One has to note that the differentiation of NWC fibres and CBD, using a fibre detection algorithm, is only reliable when CBD and NWC are sufficiently separated. This is the case for the uncycled electrode and for the inner part of the cycled electrodes. For the cycled electrodes the CBD strongly mixed with the NWC on top of the electrode, whereby no reliable differentiation was possible. However, both NWC fibres and CBD transport electrons, and collect both sulphur and Li_2_S. Therefore we treated them as one domain. Using these now segmented tomographic images, the morphology of the sulphur phase could be investigated during cycling.

**Figure 3:**
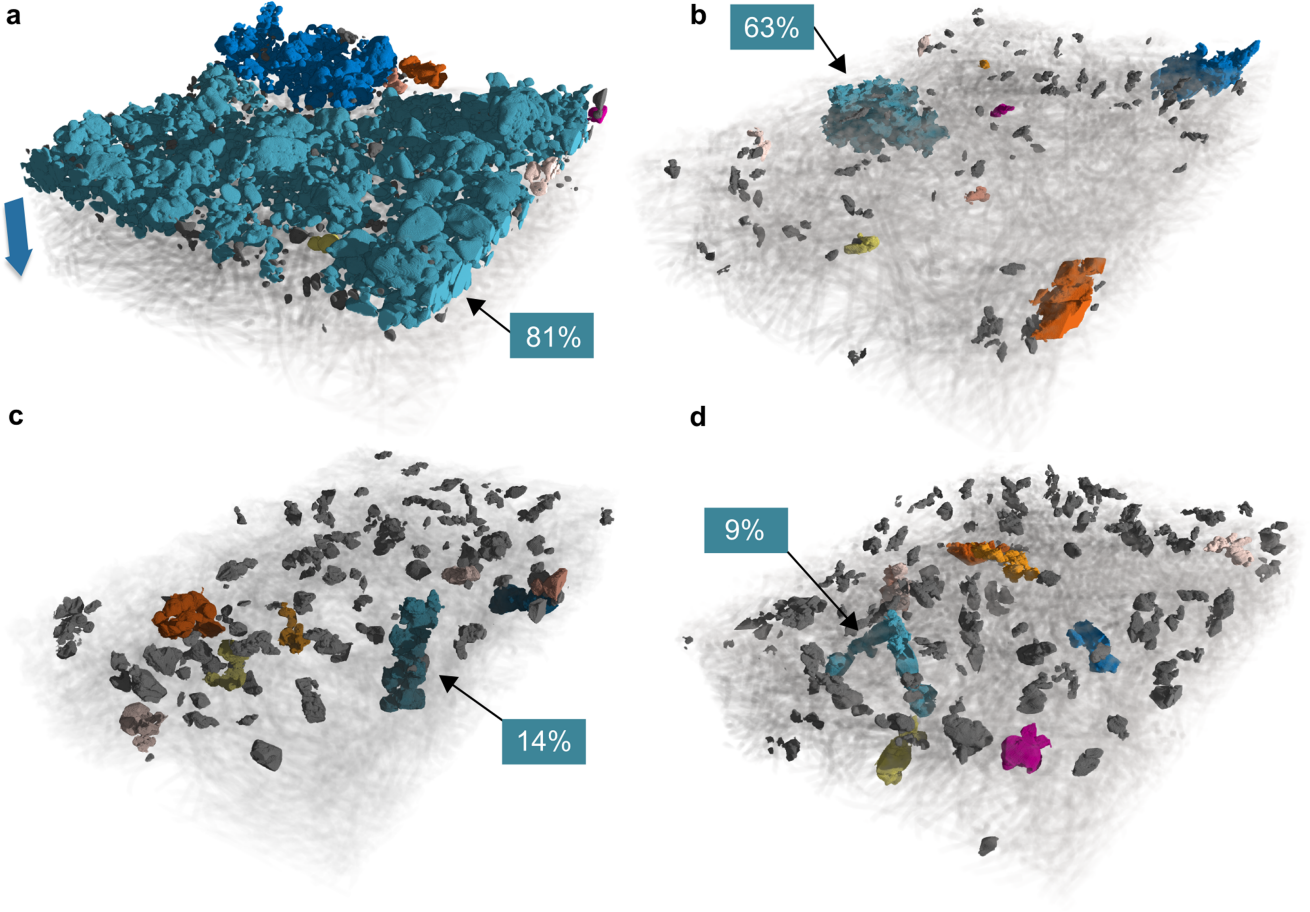
Three-dimensional distributions of sulphur particles in samples after a given number of cycles, (**a**) uncycled, (**b**) 1 cycle, (**c**) 2 cycles, (**d**) 10 cycles. Only the sulphur phase is emphasized, all other phases are set to semi-transparent, or transparent (CBD in a). The colored particles are the largest ten clusters. The grey particles are the remaining particles. The largest connected particle is marked with a black arrow and its phase fraction is given in the boxes. The amount of sulphur material clearly decreases, the particles spread, and migrate into the current collector. The spreading can also be quantified by the markedly decreasing size of the largest connected sulphur cluster (backbone) with continued cycling (Table 1). The blue arrow in a) marks the through-plane direction in which the following local calculations were done.

In Fig. 3, three-dimensional sulphur particle distributions of samples after different numbers of cycles are depicted. The coloured particles correspond to the ten largest connected sulphur clusters. Cyan indicates the largest cluster while grey marks the remaining ones. Apart from a spreading of sulphur clusters, directly pointed out by the size and phase fraction of the largest connected clusters, this representation allows for observing the migration of sulphur particles into the NWC. The spreading is also visible when plotting two-dimensional maps of sulphur volume fraction for all cycling steps. They are shown in supplementary Figure 1. It can easily be deduced from Fig. 3 that one possible explanation for the higher capacity of the NWC-based electrode is their three-dimensional structure. This structure is a potentially more stable conductive structure, with much higher porosity for larger electrolyte uptake and higher surface area, thus allowing the sulphur particles to grow again during charging.

### Sulphur loading, distribution, and particle size distribution

In all four reconstructions of Fig. 3, basic morphological parameters were extracted. Table 1 shows the area density of sulphur per μm^2^ both in voxels and mass, the surface-to-volume ratio of sulphur particles and the phase fraction of the largest connected sulphur cluster (backbone percentage), indicating the state of percolation in the sulphur domain. Generally, phase fractions reflect the occurrence percentage of a value in one phase. The largest connected cluster is called backbone. It is expressed as a fraction of the overall sulphur volume, see boxes in Fig. 3 and Table 1. From the decreasing fraction of the sulphur backbone, it can be deduced that the sulphur particles lose contact to each other during cycling.

To calculate the specific mass per area we used the densities of α- (uncycled) and β- sulphur (cycled) forms^6^,^30^. We find that the sulphur loading in the electrode decreases strongly (factor 6.8) after the first cycle. From this decrease of sulphur loading we can derive that less sulphur participates to the electrochemical process with continued cycling, which affects the discharge capacity to a large extent. It seems likely that some sulphur is lost into the electrolyte^4^, as soluble sulphur or long-chain polysulphides, or as solid products such as low solubility short-chain polysulphides. The increasing surface-to-volume ratio is a first sign of a decrease in sulphur particle size with progressing cycling.

As mentioned in the experimental section, the initial sulphur loading is approx. 3 mg cm^−^^2^, which fits well to our finding from the X-ray reconstruction, namely (3.4 ± 0.7) mg cm^−2^. This proves the good quality of the reconstruction of the sulphur phase. The scatter in Table 1 is calculated by performing the calculations in four equal domains of the reconstruction. Therefore, the scatter is a measure for homogeneity. As can be seen in Table 1, this loading significantly decreases after the first cycle from initially 3.4 mg cm^−2^ to a value of 0.5 mg cm^−2^. For further cycles up to the tenth cycle, the loading remains largely constant. However, the capacity still decreases, even if the main capacity loss was obtained after the first cycle, as can be seen in Fig. 1. For a qualitative point of view however, electrochemical response and X-ray tomography are in good accordance. However, one should note that the decrease of sulfur loading detected by X-ray tomography is severe (loading decrease by a factor of 7 after the first cycle), while the electrochemical response linked to sulphur activity is not impacted so dramatically after the first cycle. Indeed, by looking at the high voltage discharge plateau, we can have an idea of sulphur reduction activity in the cell^4^. Experimentally, between the first and second cycle, this plateau is shortened from ~200 mAh g^−1^ to 150 mAh g^−1^, which would correspond to ~25% of sulphur not being active anymore after the first cycle. Taking into account the concentration of sulphur that can be solubilized into the electrolyte (0.19 wt% in TEGDME)^31^ and the electrolyte amount introduced in the cell (150 μL), about 0.29 mg of sulphur can be found in the electrolyte in the form of soluble S_8_, *i.e.* ~5% of the total sulphur amount, which could not fully explain the decrease in sulphur loading observed upon cycling. However, we cannot exclude the formation of nano-sized sulphur particles upon cycling, since the pixel size of the used X-ray tomography is 438 nm. The nano-sized sulphur material would be electrochemical active though. Thus, the reason for such severe decay of sulfur loading upon cycling is still to be understood and must be investigated using methods with higher resolutions, e.g. focused ion beam scanning electron tomography (FIB-SEM).

We conclude that in order to quantify sulphur loadings, X-ray phase contrast tomography is suitable since the contrast of sulphur to all other phases of the electrode is high in phase contrast mode. This makes segmentation and subsequent calculation of loading for different cycling steps possible. However, as in all tomographic approaches, only features up to the resolution limit can be considered in calculations based on tomography. Sub-μm sized pores in all phases, for instance, cannot be included in our calculations.

As loading alone does obviously not fully explain capacity fading, we investigated different morphological parameters during ageing: sulphur particle sizes, particle connectivity, locations of all sulphur and carbon in the electrode, and contact area between the conducting carbon and the active sulphur.

**Figure 4:**
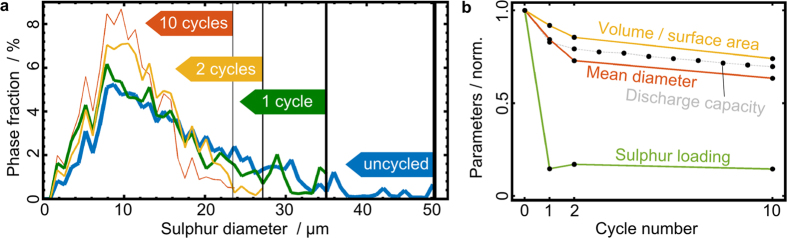
Particle size distributions and evolution of parameters compared during cycling. (**a**) Sulphur particle size distribution determined by the Delerue method^40^. The vertical lines represent the largest occurring diameter for each number of cycles. Both the largest diameter and the mean diameter, systematically decrease upon cycling. Additionally the number of larger particles decreases as a function of cycling. (**b**) Sulphur loading and different morphological parameters found in the degradation study, compared with the discharge capacity curve (grey dotted): normalized volume-to-surface area of the sulphur particles (orange), mean sulphur particle diameter (red), the sulphur loading (green). All data is normalized to the uncycled electrode parameters. We find that particle sizes decrease in a very similar fashion as the discharge capacity.

Starting with sulphur particle sizes over cycling, we plot sulphur particle diameters versus their phase fractions in Fig. 4a. It can be stated that the upper limit of diameters systematically decreases with continued cycling (vertical lines), while the maxima of all distributions remain at about 10 μm. The distance between the maximal diameters also decreases. The mean particle diameters also decrease systematically over cycling. For the uncycled electrode we obtain 16.4 μm, 13.8 μm after one cycle, 12.0 μm after 2 cycles and 10.4 μm after 10 cycles from the size distribution in Fig. 4a. This is most probably due to the fact that sulphur is being electrochemically regenerated at the positive electrode after 1, 2 and 10 cycles (with nucleation steps), and that this regrowth is less efficient over cycling.

In Fig. 4b we plot the mean sulphur particle diameters and the volume-to-surface area ratio, which is also a measure of size. They follow the capacity fading curve when normalized to the value before any cycling. In comparison to the sulphur loading, which hardly changes any more after the first cycle to the tenth cycle, the mean sulphur particle diameter does indeed roughly follow the capacity fading curve and decreases constantly. Apart from this direct measure of particle size, the volume-to-surface area ratio also follows the capacity fading curve. Since these two size-determining parameters are calculated independently, using two different methods, particle size seems to be an important parameter when it comes to explain capacity fading. However, capacity fading is thought to be a complex phenomenon, depending on passivation effects, formation of soluble active species, charge transfer resistance as well as on the contact area between sulphur and the conducting network.

**Figure 5:**
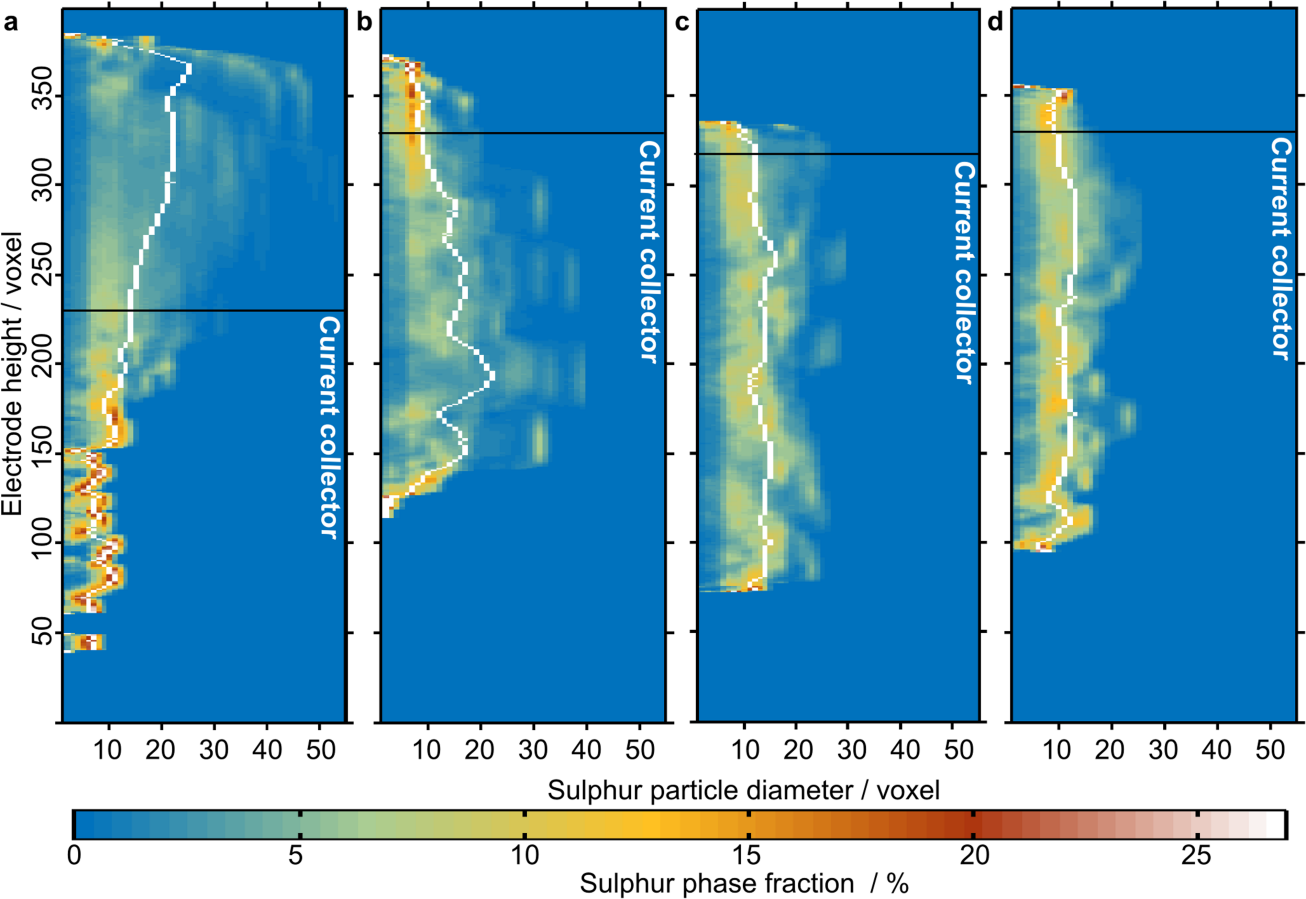
Locally resolved sulphur particle diameters in voxels (1 voxel represents (876 × 876 x876) nm^3^). The mean diameter is depicted in white. The approximate position of the current collector is shown as a black line. The colour code represents the phase fraction of each diameter. (**a**) uncycled electrode, (**b**) after 1 cycle, (**c**) after 2 cycles and (**d**) after 10 cycles. The migration into the current collector after cycling and the increasing narrowing of the distribution can be seen. The narrowing is a sign of homogenization.

**Figure 6:**
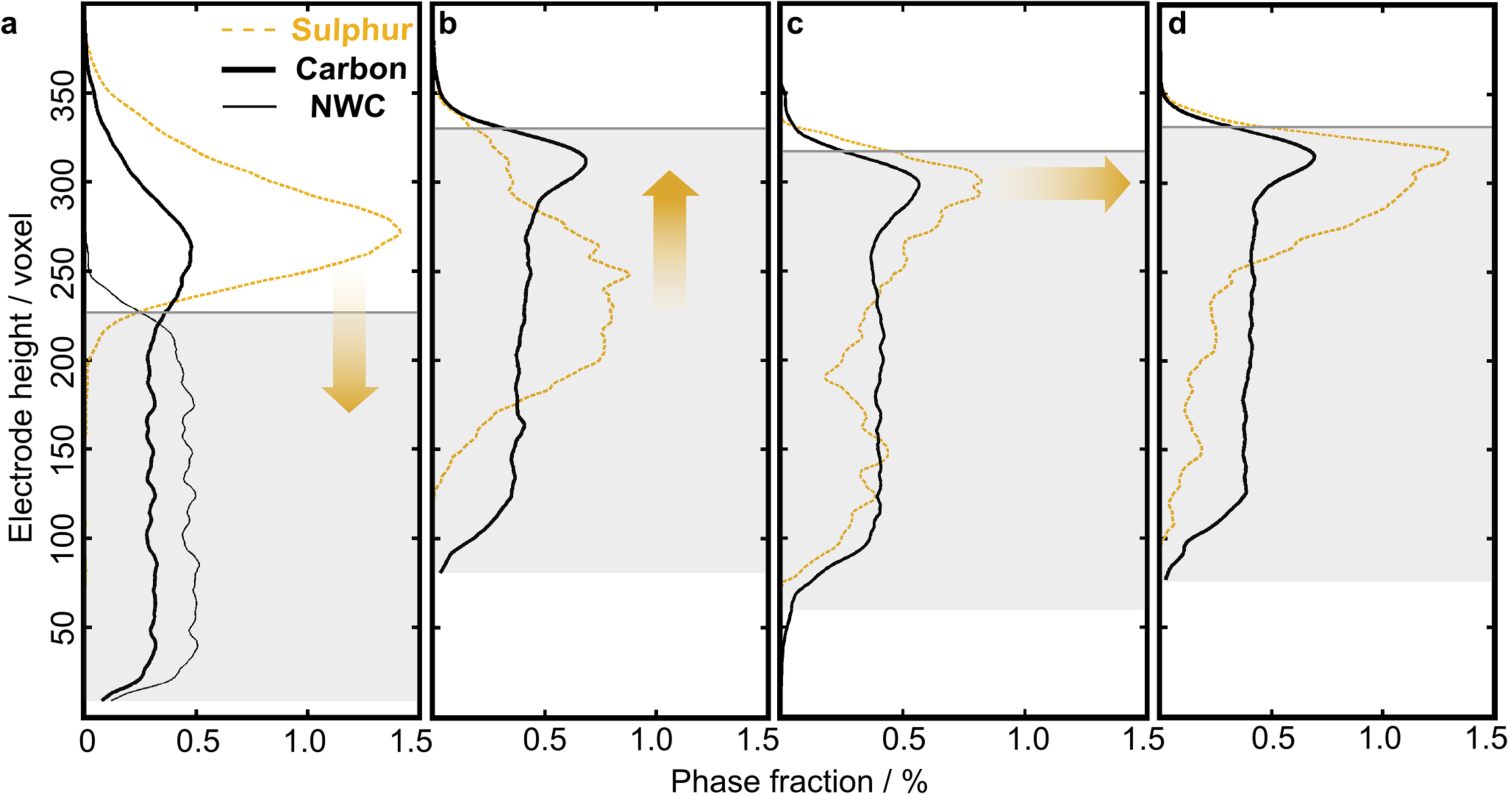
Through-plane height versus phase fraction of carbon and sulphur in the reconstructed electrodes. (**a**) uncycled electrode, (**b**) after 1 cycle, (**c**) after 2 cycles and (**d**) after 10 cycles. The dashed orange line is the sulphur phase fraction and the black line is the carbon material. For the uncycled electrode, we can clearly differentiate between the NWC and CBD. The NWC is depicted as a thin black line. The inner part of the NWC electrode is marked as a grey region. The sulphur migrates into the current collector and accumulates in the upper region of the NWC with continued cycling. This is indicated by the orange arrows.

The investigation of electrode morphology as a function of electrode height reveals further insights into the morphology of the electrode undergoing degradation. Figure 5 shows the sulphur diameters, locally resolved versus the height of the reconstructions. The colour code represents the phase fraction of a diameter at the corresponding height. From this representation, the sulphur migration into the NWC (black line marks the approx. top of the NWC by visual judgment) and a smoothening in the diameter distribution after more than 1 cycle can be seen. In the vicinity of the NWC top, we find narrow distributions (orange colour) and rather flat mean diameter curve segments. This suggests that sulphur is deposited in a rather controlled, homogeneous way after charging. From Fig. 6 we can see that the sulphur and CBD accumulate in this region.

The relative distribution of the electrical conductive material (NWC and CBD) and active material (sulphur) is of high importance for the performance of the battery, as a well conducting network is needed to facilitate sulphur utilization. In order to observe the positioning of sulphur and the carbon materials throughout the ageing process, we plotted the phase fractions of sulphur and carbon versus the through-plane direction in Fig. 6. In this representation the inner part of the NWC electrode is marked as a grey shaded region. All carbon distributions in Fig. 6 exhibit a distinct peak in the upper half of the figure. This is most probably the CBD, since the NWC itself has a very regular shape. This regularity was confirmed by checking carbon material reconstruction of the uncycled electrode, where NWC and CBD can clearly be separated (the thin black line in Fig. 6a is the NWC). When we compare the position of the carbon peak with the approximate top of the corresponding current collector, we see that for the uncycled electrode the CBD is positioned almost fully on top of the electrode. For the cycled electrodes, the CBD peak is close to the approximate top of the NWC.

The orange arrows in Fig. 6 visualize the sulphur peak evolution. We see that after 1 cycle, sulphur is primarily concentrated in the centre of the NWC. A sulphur peak, right below the NWC border line forms and grows with increased cycling. This accumulation of sulphur is in the same vertical region as the CBD peak, which infers that the main concentration of sulphur could still have contact to the CBD. This could easily be explained by the fact that CBD presents the most important specific surface area of carbon materials, with nanoparticles of about 60 m^2^g^-1^. Besides, CBD is also the electrode place where the distance between positive and negative electrodes is shorter, and the electrochemical reaction may be facilitated. The factual contact between sulphur and carbon material will be evaluated in the next section.

Our results remarkably show that sulphur particle size and distribution during ageing are not continuous, as after the first cycle drastic changes occur. However, with increasing number of cycles these changes seem to converge towards an equilibrium spatial particle distribution and mean diameter.

### Contact areas between the phases

**Figure 7:**
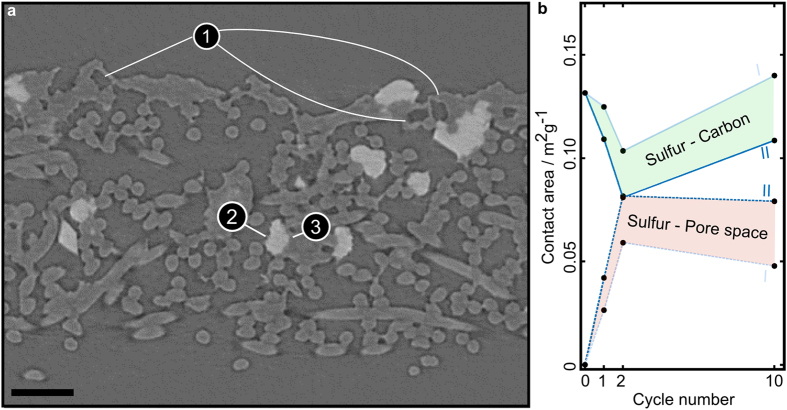
Typical cross section of an electrode after 2 cycles and contact area evolution of the phases present in a Li/S electrode. (**a**) Absorption X-ray image of the electrode after 2 cycles. The fibrous NWC can be seen. Empty CBD shells appear in the top region (1). The white particles are sulphur particles. They either have contact area with the pore space (2) or with the conducting network (3). (**b**) Contact area of sulphur and carbon material (solid lines, top), and between sulphur and pore space (dashed lines, bottom). The shaded regions represent intervals of possible values, where II represents the upper values of contact between sulphur and pore and I two lower values. They come from two different thresholds used to identify contact areas (see Method section). The scale bar is 50 μm wide.

Like discussed above, sulphur particle contact to both the electron conducting phase and electrolyte influences battery performance. In Fig. 7a, we show an X-ray tomogram after 2 cycles, to visualize contact areas. It is obvious that some sulphur particles (white) have very close contact to carbon material (3) and to pore space (2). Furthermore, empty shells on top of the electrode (1) in the size and shape of sulphur particles are visible. These shells are most probably leftovers, resulting from the initial migration of the sulphur after the first cycle. This again means that potential benefit of the CBD in this area is not used efficiently with increased cycling. These empty shells are not found in the uncycled electrode.

Figure 7b contains four curves. The upper two solid curves represent contact area between sulphur and carbon material, while the lower two curves represent contact area between sulphur and pore space. All curves are given in m^2^g^−1^. By varying the threshold of the segmentation, we are giving an interval of possible values (I underestimates the contact area of sulphur and pore space and II overestimates it). We hereby show that surface area clearly depends on the segmentation. However, within the bounds, qualitative behaviour is the same.

At contact areas between sulphur and electrolyte filled pore, the sulphur utilization will be hindered. This is because there is no conductive network available to transport the electrons, and the sulphur material itself conducts poorly. In Fig. 7b, we find that the contact area to pore space first increases and then slightly decreases after ten cycles. Contact area between sulphur and carbon potentially has a positive contribution to battery performance, as electron transport processes can occur there. Here, we consider more than just the three-phase boundary to be important, as the carbon phase contains nano-porosities^32^. In Fig. 7b, we find that sulphur-carbon contact area first decreases and again increases after 10 cycles.

Augmenting contact areas between pore and sulphur and decreasing contact area between carbon and sulphur, both contribute to capacity fading like expected. While the first three data points can be well explained, the data point generated after 10 cycles does not fit into this model. The most likely explanation for this is a changing charge transfer reaction over cycling (e.g. higher porosity of the CBD) or more severe passivation. The latter is caused by a deposition of insulating products, such as Li_2_S_2_ and Li_2_S, on the conductive surface area of the CBD somewhere between 2 and 10 cycles. We cannot also exclude capacity loss due to active species precipitation into the electrolyte upon cycling. Passivation and changing charge transfer reactions would compensate for additional contact of sulphur to carbon. Since we cannot see passivation effects directly in our X-ray tomography reconstructions, this finding could be an indirect pointer towards such effects. To our knowledge, this was never reported before. Future studies, however, must confirm these suggestions.

To conclude, we can say that a three-dimensional NWC current collector is superior to the standard two-dimensional aluminum foil current collector in terms of discharge capacity of sulphur positive electrodes. We believe that its three-dimensional structure facilitates better sulphur nucleation/growth while charging, and a better contact to the conductive network. For the superior case of the NWC-based electrode, we evaluated 3D X-ray phase contrast tomographies. We find that this technique is highly suited to differentiate between sulphur and the other electrode materials like carbon fibres and carbon-binder domain (CBD). Regarding the distribution of sulphur particles in the electrode we find that they first migrate into the current collector after one cycle and again accumulate in the top region of the electrode with increased cycling. By comparing sulphur particle sizes, distributions, contact areas, discharge capacity, and loading we find that the electrode morphology changes drastically and abruptly after the first cycle and slowly converges into an equilibrium state with continued cycling.

We calculate that sulphur loading significantly decreases after one cycle and that the loading is rather stable from there on. Since the capacity is constantly decreasing, the solid sulphur loading does not dominantly influence capacity fading after the first cycle. Surprisingly, we find that the sulphur particles get smaller as a function of cycle number, and that qualitative particle size and surface to volume ratio follow the capacity fading curve when normalized to the values before any cycling. This indicates that particle size might play an important role in explaining capacity fading by morphological parameters. However, critical parameters, such as surface passivation, transport properties, and contact areas also contribute. Contact area in particular can be calculated and interpreted by our methodological approach. Only for the first three cycling steps, favourable contact of sulphur to the electron conducting network decreases. After ten cycles, there is again more contact area to electron conducting network in spite of the decreased capacity. This can be considered an indirect sign pointing to surface passivation/deactivation, charge transfer reaction effects or loss of active species into the electrolyte.

In summary, X-ray phase contrast tomography is a powerful tool for future Li/S battery investigation, in particular to determine the degradation mechanisms and changes in electrode morphology upon cycling. However, it must be combined with methods for determining surface passivation and charge transfer mechanism and electrolyte composition in future studies.

## Methods

### Electrodes

Elemental sulphur (Refined^®^, -100mesh, Aldrich) was mixed with carbon black (SuperP^®^ Li, Timcal) and poly(vinylidene fluoride) (PVdF, Solvay) in N-methyl-2-pyrrolidinone (NMP, anhydrous, 99.5%, Aldrich). The S/C/PVDF ratio was 80/10/10 wt%, which allows producing a sulphur-rich positive electrode composition. After homogenization, the slurry was coated on a non-woven carbon paper (NWC, GDL H2315^®^, Freudenberg) and onto a 20 μm thick aluminum foil employed as current collectors and using doctor blade technique. The resulting electrodes were dried at 55 °C for 24 h, and then cut into Ø14 mm disks (1.539 cm^2^). As regards the Al-based electrodes, the coating thickness was about 100 μm so as to obtain a 20 μm thick electrode after drying, and a sulphur loading of about 1.8 mg cm^−2^. As regards the NWC-based electrodes, the sulphur loading was about 3 mg cm^−2^.

Li/S cells were assembled in an argon-filled glove box into CR2032 coin cells. A lithium metal foil was used as a negative electrode and a Celgard 2400® foil as a separator. A non-woven Viledon® separator (polypropylene-based membrane) was also added between the positive electrode and Celgard®, to provide an electrolyte reservoir. The liquid electrolyte was introduced in the cell before sealing, and was composed of LiTFSI (99.95%, Aldrich) dissolved at 1 mol L^−1^ in a mixture of tetraethylene glycol dimethyl ether (TEGDME, 99%, Aldrich, stored on molecular sieves) and 1,3-dioxolane (DIOX, anhydrous, Aldrich, stored on molecular sieves) with a 50/50 volume ratio. The Li/S cells were also cycled with another electrolyte composition, to prevent the use of a sulphur-containing lithium salt. This additional electrolyte was composed of LiNO_3_ (99.99%, Aldrich) dissolved at 0.5 mol L^−1^ in a TEGDME/DIOX 50/50 mixture. Electrochemical tests were monitored on Li/S cells using an Arbin® battery cycler, between 1.5 and 3.0 V *vs*. Li^+^/Li at C/20 rate and at room temperature. Different ageing conditions were applied to the Li/S cells in order to investigate the impact of cycling conditions on the sulphur electrode morphology and its degradation. After cycling, the Li/S cells were disassembled in an argon-filled glove box and the sulphur electrodes were recuperated. The aged electrodes were dried on a paper to prevent the use of DIOX as a washing solvent that could modify sulphur deposited layer.

### X-ray tomography

The measurements were performed at the tomography station at the BESSY electron storage ring (Helmholtz Centre Berlin, BAMline, Germany). A CdWO_4_ scintillator screen was used to convert the X-rays into visible light, which was then projected onto a CCD detector (PCO4000, 4008 × 2672 pixels). The field of view was about 1.7 × 1.2 mm^2^ with a pixel size of 438 nm. To achieve optimal image contrast the X-ray energy was set to 18.5 keV using a double multilayer monochromator that provided an energy resolution of about ΔE/E = 1.5%. Distance between sample and scintillator was set to 45 mm (phase contrast imaging mode). Overall 1800 single radiographic projection images were taken for reconstruction of the 3D images. All images were normalized with respect to the image taken without sample (flat field image). A phase retrieval algorithm based on Paganin *et al.* was applied to optimize contrast between sulphur and carbon^33^. Subsequently we coarsened the images with a factor of two, to facilitate faster calculations.

### Segmentation

For segmentation of the X-ray tomographic reconstruction, we used both information from the absorption- and the phase retrieval images. For the uncyled electrode, the contrast between carbon materials and sulphur is weaker than for the other ones. Therefore we used a combination of thresholds in absorption and phase contrast image, and assigned the phases in a process of elimination. The NWC fibres and the CBD were differentiated with a fibre detection method: It is known that the diameter of the fibres is about 9.5 μm. Therefore, the idea is to use their cylindrical shape and the known diameter to decide whether an object is a fibre or not. There are algorithms to extract fibres from 3D image data with these or similar assumptions^34^,^35^,^36^, but they would have to be extended to work for our data (e.g., not all objects are fibres). Therefore, we apply another technique that is simple and directly suitable. Considering a voxel located at the centre of a fibre, it is clear that this voxel should correspond to a (regional) minimum when considering distances inside the fibres to the pore phase. Furthermore, this minimum distance to the pore phase is in the range of about 4.75 μm. This information is used to determine whether voxels belong to fibres or not. All voxels being local minima and having the correct distance to the pore phase are selected as centres for balls. Their radius is chosen corresponding to the known fibre radius. The union of these balls forms the NWC fibres. As long as the fibres have their distinct shape, this simple model-based detection approach works quite fine.

For the cycled electrodes we averaged both absorption and phase contrast images and used an anisotropic diffusion filter to reduce noise. We thereby achieved the highest contrast between all phases. Subsequently we used the Fiji plugin statistical region merger to differentiate between pore space and solid in three dimensions^37^. For our purpose this tool is highly suitable since in our case a global threshold ends up in either a drastic underestimation of carbon material or in a spuriously segmented curb of carbon around sulphur. The region merging tool strongly reduces this effect. The sulphur phase was segmented using a global threshold in the phase contrast images (uncycled electrode) or in the absorption images (after 1, 2, and 10 cycles). Subsequently, we applied an island filter to get rid of pixel errors falsely assigned as sulphur. We further correct for the small carbon curb around sulphur by identifying high gradient regions in the averaged images. These high gradient regions are contact areas between sulphur and pore space but cannot be segmented as such in the first place. Therefor we add them to the image and delete overlaps of this region with carbon using self-programmed Matlab functions^38^. This allows us to quantify contact areas between sulphur and pore space. We made two datasets, one with a moderate threshold of the gradient image (I in Fig. 7b) and one with a low threshold (II in Fig. 7b). By applying a mean filter to the low threshold data we further enlarge the gradient regions. We make two sets of these correcting regions to show that contact area varies with segmentation but the main trends are reproduced. This also allows us to give an interval of possible values.

### Calculations

The geometrical calculations in the three-dimensional geometrical reconstructions were made using self-programmed Matlab functions (connectivity, volume fraction analysis and size analysis)^38^. The surface area calculation was done using the PoroDict package of the software GeoDict^39^. All three-dimensional images were made using VGStudio.

## Additional Information

**How to cite this article**: Zielke, L. *et al.* Degradation of Li/S Battery Electrodes On 3D Current Collectors Studied Using X-ray Phase Contrast Tomography. *Sci. Rep.* **5**, 10921; doi: 10.1038/srep10921 (2015).

## Supplementary Material

Supplementary Information

## Figures and Tables

**Table 1:** Loading, surface area to volume ratio and backbone percentage of the sulphur phase over all investigated cycling steps.

**Sulphur Parameters**	Loading / voxels*μm^−^^2^	Loading / mg*cm^−^^2^	**Surface Area:Volume / 10^5^m^−1^**	**Backbone / %**
**Uncycled**	24.8 ± 5.0	3.4 ± 0.7	2.7	81
**After 1 cycle**	3.9 ± 1.9	0.5 ± 0.2	3.0	63
**After 2 cycles**	4.5 ± 1.7	0.6 ± 0.2	3.2	14
**After 10 cycles**	3.8 ± 0.8	0.5 ± 0.1	3.7	9

The backbone percentage is the phase fraction of the largest connected cluster and is an indicator for percolation. The error was calculated by dividing the 3D structures into four compartments and average parameters of these compartments and therefore represents local distribution homogeneity.
