# Supplementary material for: Degradation of Li/S Battery Electrodes On 3D Current Collectors Studied Using X-ray Phase Contrast Tomography
Source: Sci Rep. 2015 Jun 4;5:10921. doi: 10.1038/srep10921 (PMC4455252; doi:10.1038/srep10921)
Supplement: Supplementary Information [file srep10921-s1.pdf]

Degradation of Li/S Battery Electrodes On 3D Current Collectors Studied Using X-ray Phase Contrast Tomography

L. Zielke[1*], C. Barchasz[2], S. Waluś[2,3,4], F. Alloin[3,4], J.-C. Leprêtre[3,4], A. Spettl[5], V. Schmidt[5], A. Hilger[6], I. Manke[6], J. Banhart[6], R. Zengerle[1,7] and S. Thiele[1,8]

In the supplementary Figure 1s we show the sulphur volume fraction in the whole cathode thickness from above. The mentioned spreading of particles can be seen from b) to d).

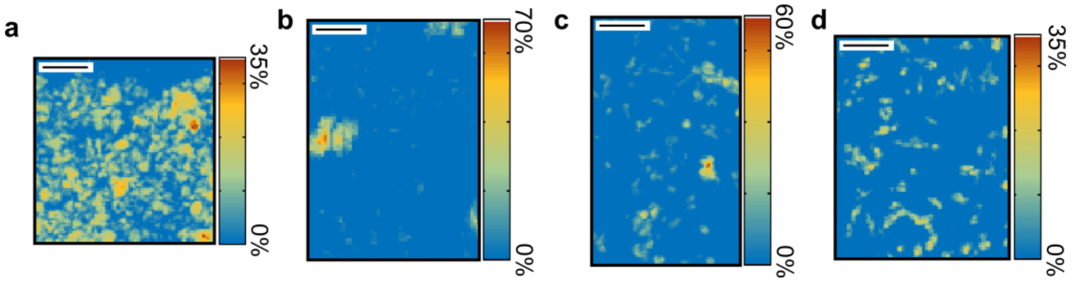

Figure 1s Projected sulphur volume fractions in the four investigated positive electrodes, as seen from above. (a) uncycled electrode, (b) after 1 cycle, (c) after 2 cycles and (d) after 10 cycles. One pixel represents the volume fraction of sulfur in a corresponding volume of 8.8 µm x 8.8 µm x local thickness of the cathode. The spreading of sulphur material during cycling can be seen (b-d). The scale bar represents 200 µm.
